# The evolving landscape of *Staphylococcus aureus* immune evasion: a data-driven atlas over two decades

**DOI:** 10.3389/fimmu.2026.1862459

**Published:** 2026-07-16

**Authors:** Yize Fan, Shu Wang, Xingyu Zhu, Xiaotong Zhang, Ce Zhang, Chengshuai Yang, Qiuting Wang, Jiadi Zhao, Dingyuan Guo, Luyuan Ma, Qian Zhao, Caiyan Zhao, Jiajia Lu, Chuan Shen

**Affiliations:** 1 Burns & Wound Healing Center, Hebei Medical University Third Hospital, Shijiazhuang, China; 2 Department of Infectious Disease, Hebei Medical University Third Hospital & Clinical Research Center for Infectious Disease of Hebei Province & Hebei Key Laboratory for Diagnosis, Treatment, Emergency Prevention and Control of Critical Infectious Diseases, Shijiazhuang, China; 3 Department of Laboratory Medicine, The First Hospital of China Medical University, Shenyang, China; 4 Office of Quality Management and Control in Healthcare, Hebei Medical University Third Hospital, Shijiazhuang, China; 5 Department of Orthopedic Trauma, Shanghai Fourth People’s Hospital, School of Medicine, Tongji University, Shanghai, China

**Keywords:** bibliometrics, biofilm, immune evasion, immunopathogenesis, immunotherapy, *Staphylococcus aureus*

## Abstract

**Background:**

*Staphylococcus aureus* (*S. aureus*) has emerged as a significant global public health threat, owing to its complex immune evasion strategies. Despite growing research output in this field, a comprehensive, data-driven bibliometric analysis delineating its global landscape, research hotspots, and translational gaps remains lacking.

**Methods:**

Publications from 2006 to 2025 were retrieved from the Web of Science Core Collection (WOSCC) and Scopus databases, which formed the core dataset for bibliometric analysis. Clinical trial data were independently supplemented from PubMed to assess translational progress. Multidimensional bibliometric analyses and visualization were performed using R-bibliometrix, VOSviewer and CiteSpace.

**Results:**

A total of 1,374 eligible publications were included, with an average annual growth rate of 15.71%, indicating a rapidly expanding field. The research landscape exhibits a hierarchical, stepwise distribution dominated by the United States, with active contributions from multiple countries. Utrecht University and the University of California system were the most prolific institutions. Frontiers-series journals serve as the major publication outlets. Knowledge structure analysis indicates a paradigm shift from fundamental immune mechanisms toward antibiotic resistance, biofilm biology, and immunometabolic regulation. Integration of PubMed clinical data revealed a critical translational gap, as despite advances in mechanistic research, most candidate vaccines and monoclonal antibody therapies failed to meet primary clinical endpoints.

**Conclusion:**

Research on *S. aureus* immune evasion is transitioning from foundational mechanistic studies toward multi-omics integration and clinical translation. Future efforts should transcend single-target strategies, focusing on novel therapeutic approaches grounded in immunometabolic modulation and precision treatment regimens.

## 1 Introduction

*Staphylococcus aureus* (*S. aureus*), a highly adaptable and ubiquitously distributed pathogen, has long represented a major threat to global public health (1). Data from the Global Burden of Disease study indicate that *S. aureus* is not only a leading cause of bacterial mortality but also a key driver of multidrug-resistant infections worldwide (2). Its notable evolutionary plasticity allows it to stably colonize mucosal surfaces while breaching epithelial barriers to enter the bloodstream, resulting in systemic dissemination and a spectrum of life-threatening conditions, including infective endocarditis, osteomyelitis, and device-associated infections (3–5).

Accumulating evidence indicates that the high lethality of *S. aureus* is largely driven by its sophisticated immune evasion strategies. Unlike pathogens that passively withstand host defenses, *S. aureus* actively modulates and reshapes host immune networks. During immune recognition, it attenuates innate immune activation by disrupting pattern recognition receptor signaling, including Toll-like receptor 2 (TLR-2) pathways (6, 7). In the effector phase, the bacterium utilizes virulence factors such as α-hemolysin and leukocidins to compromise immune cell function and suppress neutrophil activity, thereby diminishing pathogen clearance (8, 9). Furthermore, *S. aureus* induces immunometabolic remodeling, promoting the expansion of myeloid-derived suppressor cells and increasing interleukin-10 (IL-10) production through metabolic intermediates such as lactate, ultimately establishing an immunosuppressive microenvironment (10, 11).

The extraordinary redundancy and multifaceted nature of these immune evasion mechanisms have resulted in a highly fragmented research landscape, with investigations scattered across microbiology, immunology, infectious diseases, and translational medicine disciplines (12). This fragmentation creates critical barriers to identifying consensus mechanisms, resolving conflicting findings, and translating preclinical discoveries into effective clinical interventions.

Previous bibliometric studies typically based on a single database (13, 14), face inherent limitations due to restricted coverage and infrequent updates, which hinder a comprehensive view of the field. Additional shortcomings include: (1) truncated timeframes that do not capture the full two-decade evolution of research; (2) insufficient integration of clinical trial data, vital for assessing translational progress; (3) basic thematic analyses that fail to differentiate between foundational mechanisms and emerging therapeutic targets; and (4) inadequate cross-database validation, which can introduce systematic biases in citation and collaboration network analyses. To address these limitations, this study establishes a clearly defined two-tiered analytical framework. The core bibliometric analysis is conducted exclusively using the Web of Science Core Collection (WoSCC) and Scopus databases, while PubMed is independently queried to gather clinical trial data for evaluating translational progress. This integrated approach leverages large-scale quantitative mapping of the global research landscape alongside targeted clinical outcome analysis, potentially facilitating a systematic and comprehensive evaluation of *S. aureus* immune evasion research over the past two decades.

## 2 Materials and methods

### 2.1 Data sources and cleaning

The data for this study were derived from two major scholarly databases, the WOSCC and Scopus, to ensure both comprehensiveness and robustness of the results. The retrieval window spanned from January 1, 2006, to December 31, 2025, restricted to English-language publications and limited to the document types Article and Review. Boolean logic operators were employed to combine subject-specific terms related to *S. aureus* and immune evasion. The search strategies were as follows: For the WoSCC, the search was conducted using the following query: TS = (“*Staphylococcus aureus*” OR “*S. aureus*” OR “*Staph aureus*”) AND (“immune evasion” OR “immune escape” OR “immune suppress*” OR “immune avoid*” OR “host immune response evasion”). For Scopus, the search strategy employed was: (TITLE-ABS-KEY (“*Staphylococcus aureus*” OR “*S. aureus*” OR “*Staph aureus*”)) AND TITLE-ABS-KEY (“immune evasion” OR “immune escape” OR “immune suppress*” OR “immune avoid*” OR “host immune response evasion”).

**Figure 1:**
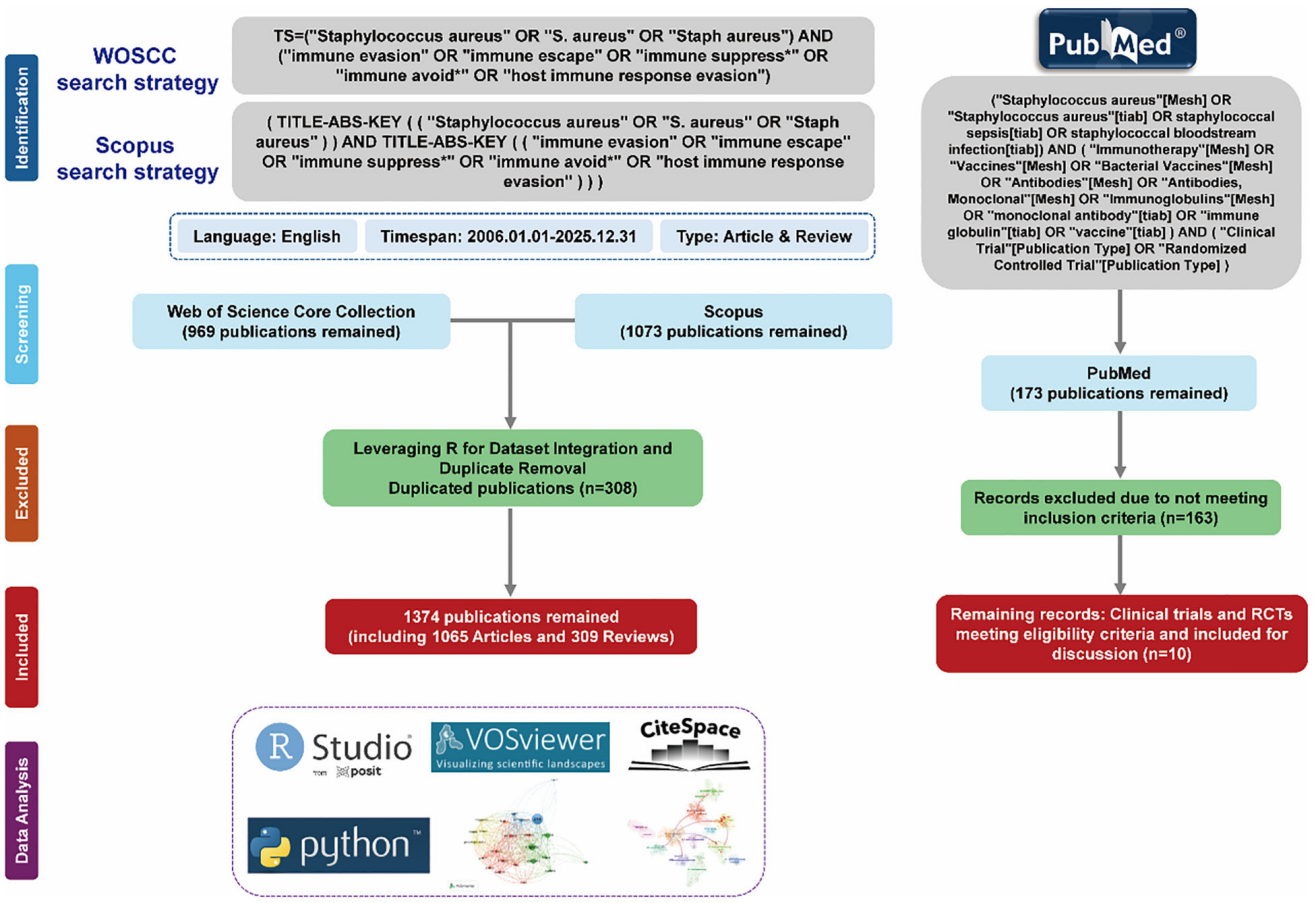
Preferred reporting items for systematic reviews and meta-analyses flow diagram illustrating the literature search and screening process. This diagram depicts the identification and screening workflow for studies regarding *S. aureus* immune evasion across the Web of Science Core Collection (WOSCC), Scopus, and PubMed databases.

The initial search retrieved 969 records from WoSCC and 1,073 records from Scopus. Data integration and duplicate removal were performed using an R-based workflow, in accordance with previously established bibliometric methodologies (15, 16) Records were systematically cross-matched based on title, author information, and publication year, resulting in the exclusion of 308 duplicates and yielding a final dataset of 1,374 unique publications, including 1,065 original articles and 309 reviews (**Figure 1**). To complement the clinical perspective, relevant records were independently retrieved from PubMed.

Screening was conducted according to pre-defined inclusion and exclusion criteria by three researchers (Fan YZ, Wang S, and Wang QT) independently, with discrepancies resolved through discussion and calculation of inter-rater agreement (Kappa = 0.88, 95% CI [0.82, 0.94]). Detailed search strings for all three databases are provided in **Supplementary Table 1**. Titles, abstracts, and keywords of all included publications formed the corpus for subsequent analyses. During data cleaning, text preprocessing was carried out using R packages, standardizing content by removing non-alphabetic characters, punctuation, and extraneous whitespace to minimize noise from formatting inconsistencies. From an initial set of 173 candidate studies, 10 clinical trials and randomized controlled trials (RCTs) were ultimately included in the analysis (**Figure 1**). From an initial set of 173 candidate studies, 10 clinical trials and randomized controlled trials (RCTs) were ultimately included in the analysis (**Figure 1**). Eligible studies were limited to clinical trials and RCTs meeting all the following criteria, which enrolled adult patients, pediatric patients and very low birth weight infants diagnosed with *S. aureus* infection or identified at high risk of *S. aureus* infection, implemented targeted therapeutics against specific *S. aureus* antigens (i.e., monoclonal antibodies, polyclonal immunoglobulins and vaccines) with placebo or standard of care (SoC) served as controls, and reported no less than one clinically efficacy endpoint, including composite clinical outcomes, time to bacteremia clearance, time to fever resolution, as well as the incidence of *S. aureus* infection, secondary pneumonia and bacterial colonization. By contrast, studies were excluded if they belonged to non-randomized designs (literature reviews, case reports and observational studies), enrolled populations with unclear *S. aureus* infection status or undefined high-risk characteristics, adopted interventions untargeted at *S. aureus* specific antigens, lacked placebo or SoC control groups, or failed to extract valid clinical outcome data for pooled analysis (**Supplementary Table 2**).

### 2.2 Bibliometric analysis

A comprehensive suite of bibliometric tools was employed to perform multidimensional quantitative analyses on the included publications, covering metrics such as geographic and institutional distribution, collaboration networks, journal co-occurrence, and citation structures. Descriptive statistics—including annual publication trends, core author identification, journal distribution, and citation analysis—were generated using the bibliometrix package (v5.0) in R (v4.4.5). Original network data were extracted, and thematic evolution bubble matrices were constructed to visualize shifts in research focus over time.

Network analyses of authors, countries, and institutions were conducted using VOSviewer (v1.6.20), employing the Fruchterman-Reingold force-directed layout. Association strength normalization was applied to mitigate scale effects, and community detection was performed using the Walktrap algorithm. To enhance the robustness of thematic clustering, an additional manual validation step was performed. Two independent reviewers screened and refined clustered keywords to ensure biological relevance to *S. aureus* immune evasion. Discrepancies were resolved through consensus discussion. CiteSpace (v6.2.R1) was utilized for keyword clustering, keyword burst detection, and citation burst analysis, with a temporal slicing interval of one year and stringent node selection parameters (g-index, k = 25; LRF = 2.5; L/N = 10; LBY = 5; e = 1.0). Raw outputs from all software were imported into Python (v3.14) and harmonized using pandas, matplotlib, seaborn, and networks for standardized formatting and enhanced visualization (17–19).

## 3 Results

### 3.1 General characteristics of publications and publication trends

A total of 1,374 publications were included in the analysis, comprising 1,065 original research articles and 309 reviews, spanning 452 journals. The temporal coverage of the dataset extended from 2006 to 2025, with an average annual growth rate of 15.71%, reflecting a notable upward trajectory in research activity within this field. The mean number of citations per document was 48.57, indicating substantial scholarly influence (**Table 1**).

**Table 1:** Comparison of general literature characteristics across databases (WOSCC, SCOPUS, and merged dataset).

Description	WOSCC	Scopus	WOSCC+Scopus
Timespan	2006:2025	2006:2025	2006:2025
Sources (Journals, Books, etc.)	303	390	452
Documents	969	1073	1374
Annual Growth Rate %	14.51	17.83	15.71
Document Average Age	7.95	7.46	7.82
Average citations per doc	46.33	46.97	48.57
References	38568	7469	42030
Keywords Plus (ID)	2615	8997	7935
Author’s Keywords (DE)	1990	2269	2755
Authors	5366	6016	7662
Authors of single-authored docs	14	25	31
Single-authored docs	20	31	40
Co-Authors per Doc	7.49	7.41	7.22
International co-authorships %	33.75	32.53	31.51
article	797	826	1065
review	172	247	309

**Figure 2:**
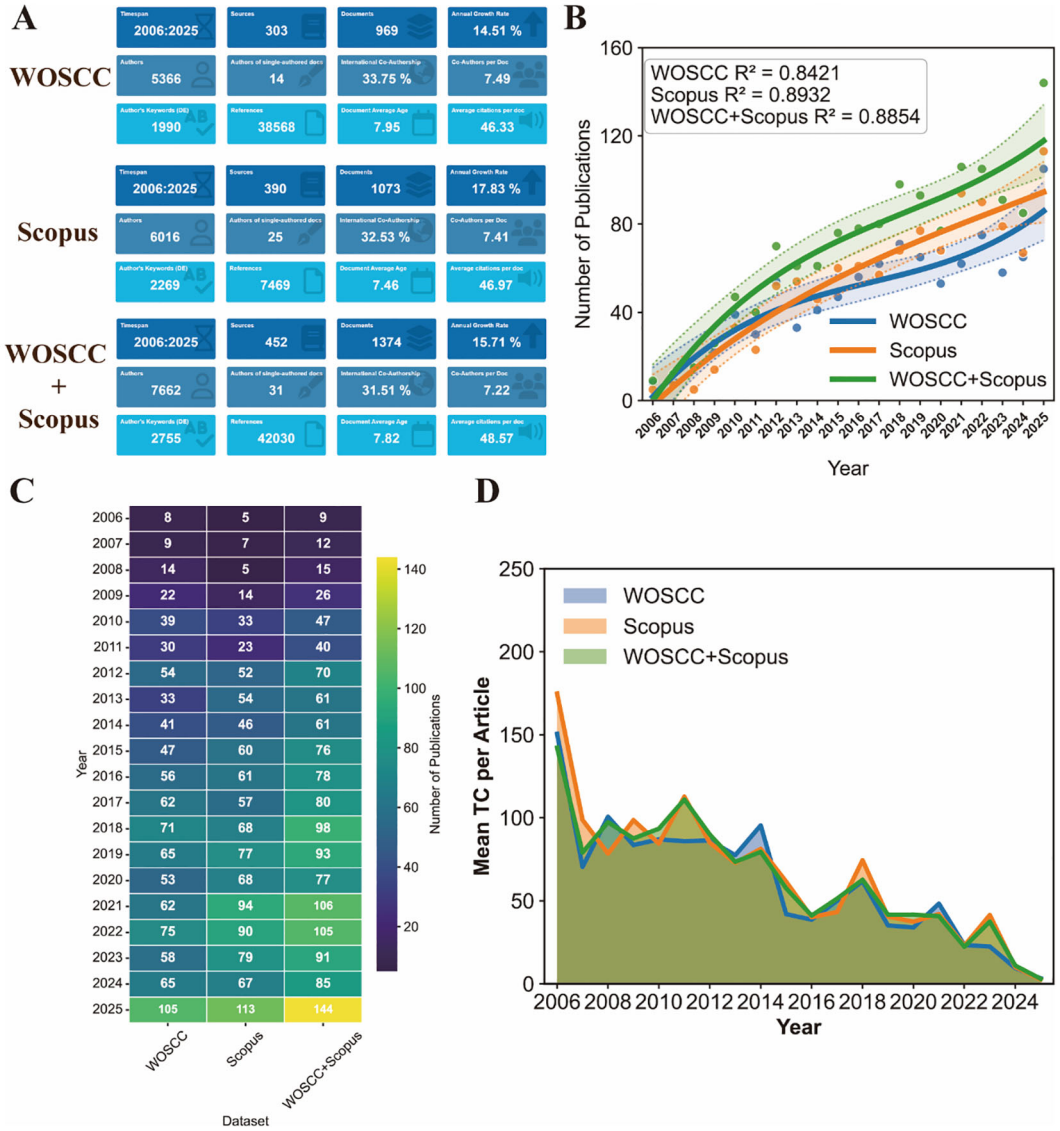
Analysis of annual publication trends and citation dynamics. **(A)** Comparison of general metrics and publication trends across WOSCC, Scopus, and the integrated WOSCC+Scopus dataset. **(B)** Statistics on annual publication output from 2006 to 2025, featuring a third-degree polynomial regression curve (R2 = 0.8854) that highlights the exponential growth phase of the field. **(C)** Quantitative comparison of annual publication volumes among the three datasets. **(D)** Analysis of annual citation counts, demonstrating a time-lag effect where earlier publications (e.g., 2006) exhibit higher cumulative citation counts compared to recent years (e.g., 2024).

Authorship analysis identified 7,662 unique contributors, including 31 single-author publications. The average number of co-authors per paper was 7.22, and internationally co-authored articles constituted 31.51% of the dataset (**Figure 2A**; **Table 1**). Ordinary least squares (OLS) fitting using a cubic polynomial regression model demonstrated a high goodness-of-fit (R^2^ = 0.8854) for the combined WoSCC and Scopus dataset, consistent with a pronounced acceleration in publication output since 2006 (**Figure 2B**).

Temporal examination of annual publication counts further illustrated this trend. Between 2006 and 2010, yearly output was relatively modest, generally ranging from single digits to low tens, followed by a period of steady growth. After 2019, when 93 publications were recorded, output displayed a fluctuating upward trajectory, culminating in a peak of 144 publications in 2025, suggesting continuous growth of research outputs in the field (**Figure 2C**). Citation analysis revealed a clear temporal lag effect (**Figure 2D**; **Supplementary Table 3**). Early publications benefited from a longer citation accumulation window and thus exhibited higher citation counts—for example, papers published in 2006 averaged 142.22 citations. In contrast, more recent publications, such as those from 2024, have not yet reached their full citation potential, averaging only 10.96 citations. This progressive decline in mean citations per year reflects the typical dynamics of citation accrual observed in scientific literature (**Figure 2D**; **Supplementary Table 3**).

### 3.2 Core contributors and source distribution patterns

**Figure 3:**
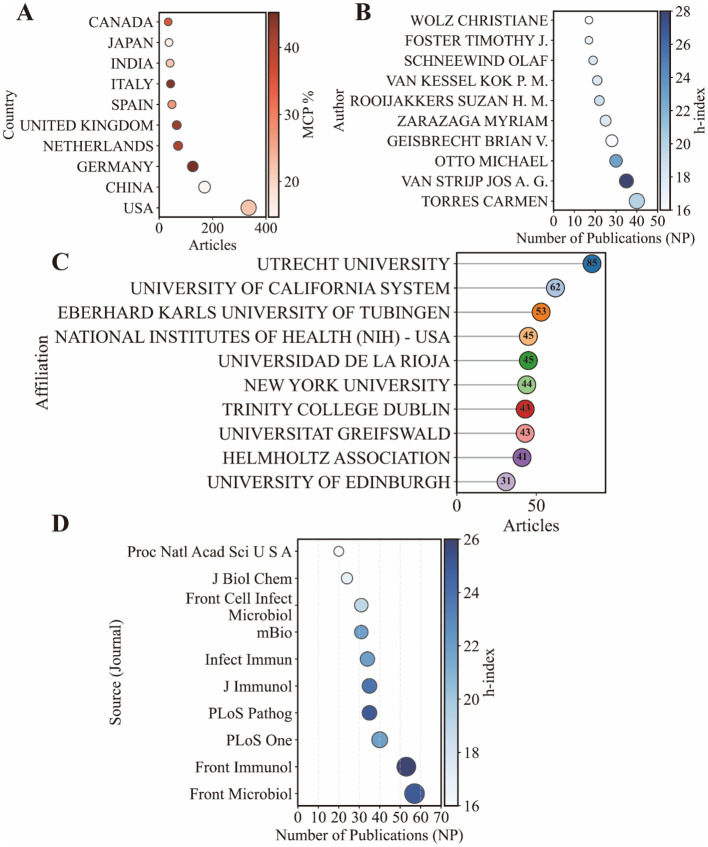
Landscape of core contributors and source distribution. **(A)** Lollipop chart of the top ten countries by publication output. Circle size represents the Multiple Country Publication percentage (MCP%). **(B)** Bubble plot of the top ten authors ranked by H-index, with bubble size corresponding to total citation counts. **(C)** Lollipop chart of the top ten institutions by publication output. **(D)** Bubble plot of the top ten journals ranked by H-index, highlighting the dominance of the Frontiers series (e.g., Frontiers in Immunology, Frontiers in Microbiology) in disseminating research outcomes.

Analysis of national contributions revealed that the United States led the field with 335 publications (24.4%), suggesting it functions as a major hub of research activity, followed by China with 170 publications (12.4%) (**Table 2**). International collaboration intensity varied considerably among countries: Germany (45.2%), the Netherlands (40.8%), and the United Kingdom (42.4%) exhibited high proportions of internationally co-authored publications (MCP%), highlighting a strong reliance on cross-border partnerships. In contrast, China (14.7%) and the United States (22.4%) showed relatively lower MCP%, indicative of well-established, largely independent domestic research infrastructures (**Figure 3A**; **Table 2**).

**Table 2:** Analysis of the top ten countries by publication output and collaboration intensity (WOSCC+SCOPUS).

Country	Articles	Articles %	SCP	MCP	MCP %
USA	335	24.4	260	75	22.4
CHINA	170	12.4	145	25	14.7
GERMANY	126	9.2	69	57	45.2
NETHERLANDS	71	5.2	42	29	40.8
UNITED KINGDOM	66	4.8	38	28	42.4
SPAIN	48	3.5	35	13	27.1
ITALY	43	3.1	24	19	44.2
INDIA	41	3	32	9	22
JAPAN	37	2.7	31	6	16.2
CANADA	34	2.5	22	12	35.3

Data generated using the R package ‘bibliometrix’. MCP% indicates the proportion of a country’s publications involving international co-authorship.

High-impact scholars and core institutions were highly concentrated. VAN STRIJP JOS A. G. ranked first in terms of academic metrics, with an h-index of 28 and 2,460 total citations (TC), consistent with a strong per-article impact (**Figure 3B**; **Table 3**). OTTO MICHAEL (h-index 23, TC 4,218) and TORRES CARMEN (h-index 20, TC 1,016) represented the next tier of central contributors (**Figure 3B**; **Table 3**). Utrecht University (85 publications) and the University of California system (62 publications) were the most prolific institutions, often closely associated with these leading scholars, forming a tightly coupled “top scholar–core institution” network (**Figure 3C**).

**Table 3:** Top ten authors ranked by academic quantitative metrics (WOSCC+SCOPUS).

Author	h_index	g_index	m_index	TC	NP	PY_start
VAN STRIJP JOS A. G.	28	35	1.333	2460	35	2006
OTTO MICHAEL	23	30	1.211	4218	30	2008
TORRES CARMEN	20	31	1.333	1016	40	2012
ROOIJAKKERS SUZAN H. M.	19	22	0.905	1632	22	2006
SCHNEEWIND OLAF	18	19	0.947	2452	19	2008
VAN KESSEL KOK P. M.	18	21	0.857	1615	21	2006
ZARAZAGA MYRIAM	18	25	1.2	794	25	2012
FOSTER TIMOTHY J.	17	17	0.81	2668	17	2006
GEISBRECHT BRIAN V.	16	28	0.8	1458	28	2007
WOLZ CHRISTIANE	16	17	0.762	1097	17	2006

Examination of source journal distribution indicated that the Frontiers journal series accounted for a significant proportion of the publication landscape (**Figure 3D**; **Table 4**). Frontiers in Immunology ranked highly with 53 publications and an h-index of 26, ranking high in publication volume in this field. Frontiers in Microbiology followed closely, contributing 57 publications with an h-index of 25. Although PLoS Pathogens published fewer articles (35), its h-index of 25 and early establishment (first publication year = 2008) reflected a longstanding presence in the field. Proceedings of the National Academy of Sciences (PNAS), despite a smaller output (20 publications), maintained a high h-index (16), potentially reflecting rigorous selection standards and strong scholarly reputation. Overall, relevant findings are widely distributed on open-access platforms for broad visibility, while high-level breakthroughs continued to appear in top-tier multidisciplinary journals, resulting in a multilayered and multidimensional academic communication landscape.

**Table 4:** Top ten journals ranked by academic quantitative metrics (WOSCC+SCOPUS).

Source	h_index	g_index	m_index	TC	NP	PY_start
Front Immunol	26	45	2	2115	53	2014
Front Microbiol	25	42	2.273	1868	57	2016
PLoS Pathog	25	35	1.316	2594	35	2008
J Immunol	24	35	1.2	3177	35	2007
Infect Immun	22	34	1.158	1340	34	2008
mBio	22	31	1.467	2176	31	2012
PLoS One	22	38	1.222	1490	40	2009
Front Cell Infect Microbiol	19	31	1.267	1108	31	2012
J Biol Chem	17	24	0.81	938	24	2006
Proc Natl Acad Sci U S A	16	20	0.8	1253	20	2007

### 3.3 Analysis of author, institution, and country output

**Figure 4:**
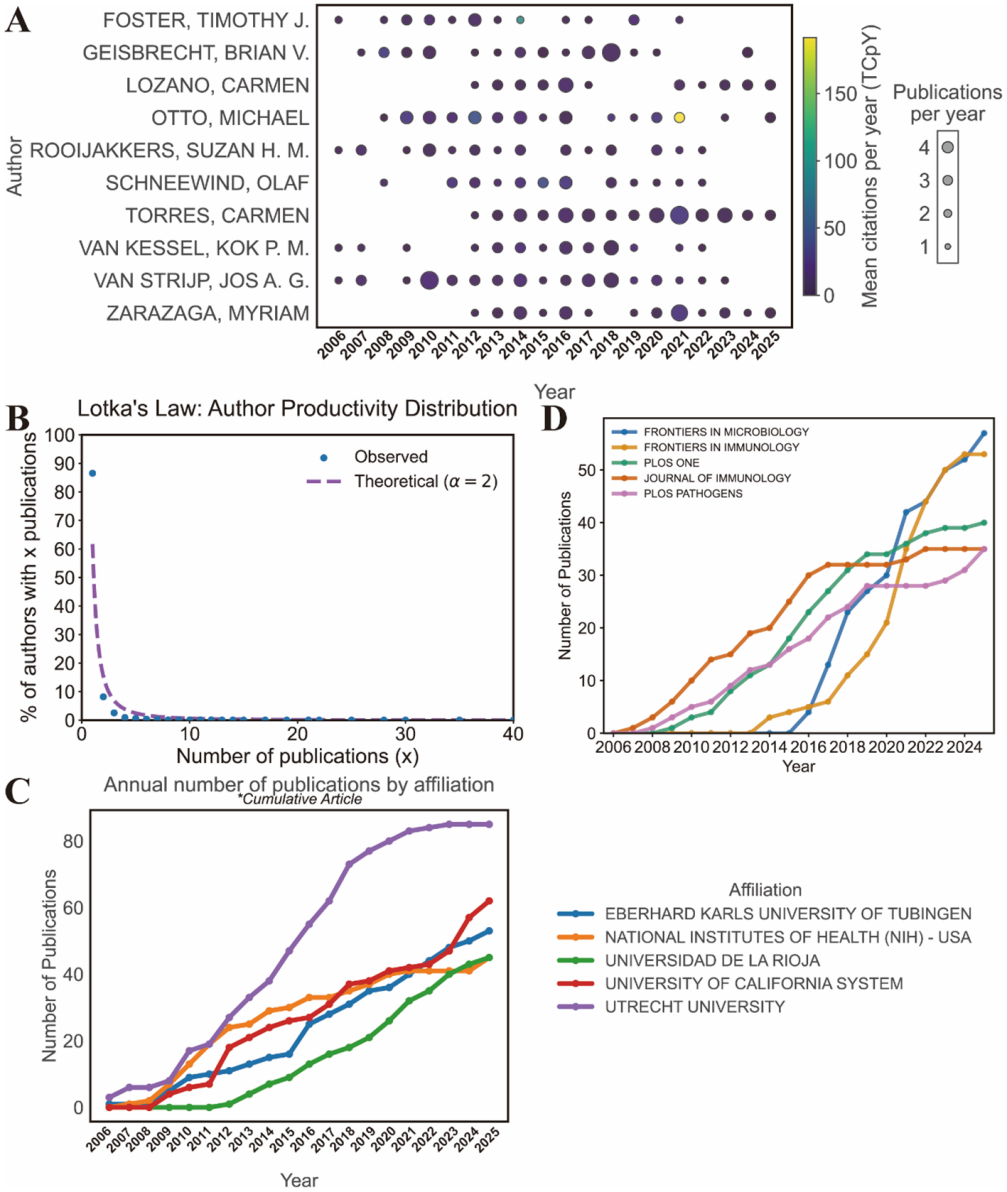
Temporal analysis of productivity among authors, institutions, and journals. **(A)** Annual publication trends of core authors. **(B)** Analysis of author productivity distribution based on Lotka’s Law, revealing extreme scattering where approximately 86.5% of authors contributed only a single paper. **(C)** Cumulative publication output trends for the top five institutions. **(D)** Cumulative publication output trends for the top five journals.

A temporal analysis of author, institutional, and journal outputs was conducted to elucidate evolving patterns and the concentration of scholarly productivity within the field (**Figure 4**). Research output appears to be highly concentrated among a small group of leading scholars, who have maintained sustained productivity over time. TORRES CARMEN reached a peak of six publications in 2021 and continued to publish at a high rate through 2025. OTTO MICHAEL produced two highly cited papers in 2021, reflecting enduring academic influence. Two papers published by OTTO MICHAEL in 2021 received a large number of citations. Other key contributors, including FOSTER TIMOTHY J., SCHNEEWIND OLAF, and ROOIJAKKERS SUZAN H. M., also made substantial contributions to the core literature during the latter half of the 2010s (**Figure 4A**).

Analysis based on Lotka’s Law further substantiates this distribution: approximately 86.5% of authors contributed only a single paper, substantially exceeding the theoretical expectation of 62.6%. This indicates extreme dispersion of productivity, with a small group of scientists accounting for the majority of high-impact publications (**Figure 4B**).

At the institutional level, Utrecht University ranked first in cumulative publications. Its publication output increased steadily from 2006 and reached a cumulative total of 85 publications by 2025, establishing its position as a core institution in the field. Eberhard Karls University of Tübingen follows closely with a similarly robust growth trajectory, reaching 53 cumulative publications by 2025. The National Institutes of Health (NIH, USA) and the University of California System rank third and fourth, with cumulative outputs of 45 and 62 publications, respectively. Universidad de La Rioja started related research in 2012, and its publication volume grew rapidly, with over 40 cumulative articles within five years (**Figure 4C**).

Regarding journal dissemination, open-access journals have published a greater number of research findings worldwide, which may enhance the accessibility and sharing of scientific data. Frontiers in Microbiology showed a remarkable increase in annual publications rising from four in 2016 to 57 in 2025, while Frontiers in Immunology increased from three publications in 2014 to 53 in 2025. In comparison, traditional authoritative journals such as PLOS ONE and Journal of Immunology maintained relatively stable outputs (40 and 35 publications in 2025, respectively). PLOS Pathogens, although producing fewer articles (35 in 2025), consistently upheld high academic standards and exerted significant scholarly influence (**Figure 4D**).

### 3.4 Collaborative networks among authors, countries, and institutions

**Figure 5:**
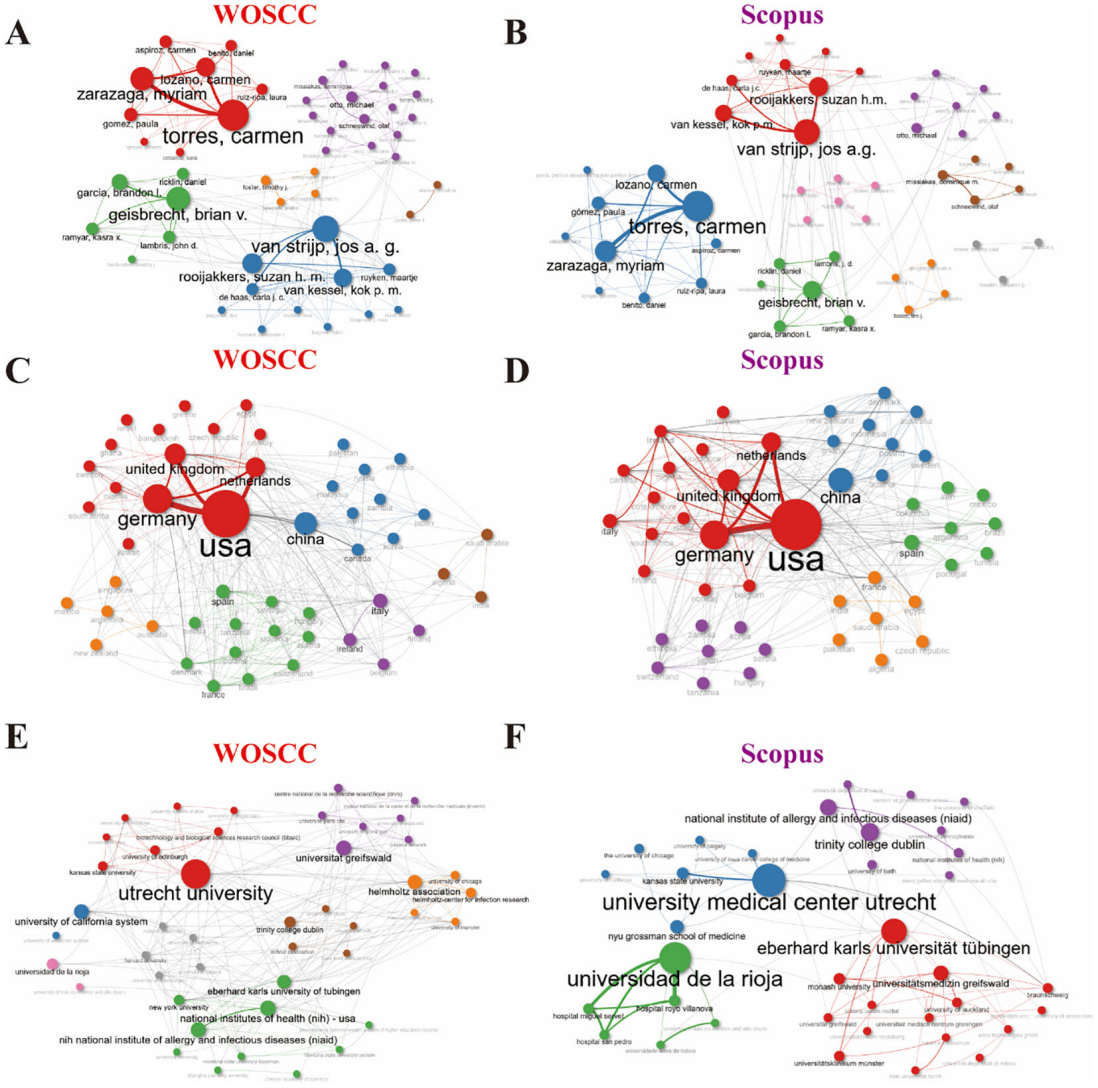
Comparative analysis of collaboration networks. **(A, B)** Author collaboration networks. **(C, D)** Country collaboration networks. **(E, F)** Institution collaboration networks.

To elucidate the structural characteristics and identify key actors within the field, collaborative networks were constructed for authors, countries, and institutions using the WoSCC and Scopus datasets via the R bibliometrix package (**Figure 5**).

Analysis of author collaborations revealed substantial concordance between the two databases in identifying core collaborative structures. Across both datasets, a central cluster comprising TORRES CARMEN, VAN STRIJP JOS A. G., and GEISBRECHT BRIAN V. was consistently observed (**Figures 5A, B**). These authors occupy high-centrality positions within the network, with overlapping connections and well-defined community structures, highlighting the stability of principal research teams and their internationally recognized scholarly influence. The Scopus network exhibits slightly greater heterogeneity, with a modestly higher number of peripheral nodes and sparser connections, which may reflect occasional collaborations by non-core authors that are not captured in WoSCC. Nevertheless, these differences do not alter the overarching network structure (**Figure 5B**).

At the national level, the United States, Germany, the United Kingdom, and the Netherlands appear to form a close international collaboration group, and researchers from Western Europe and North America have produced a large number of studies in this field (**Figures 5C, D**). The geographic distribution of cross-national partnerships is highly consistent across databases, underscoring the sustained scientific leadership of Western Europe and North America in this domain.

Institutional networks reveal Utrecht University and Eberhard Karls University of Tübingen as central nodes in both datasets, supporting their status as major research institutions. Other prominent institutions, including the U.S. National Institutes of Health (NIH), Universidad de La Rioja, and Universität Greifswald, were also consistently identified as key contributors, reflecting a stable global distribution of leading research centers. Observed heterogeneity may primarily arises from limitations in Scopus’s institutional name standardization. For example, Scopus treated “University Medical Center Utrecht” and “Utrecht University” as separate entities, whereas WoSCC consolidated them into a single node, providing more consistent data normalization (**Figures 5E, F**). Furthermore, the Scopus network displays slightly sparser connectivity and captures institutions such as NYU Grossman School of Medicine and Trinity College Dublin, which are less prominent in WoSCC, illustrating that nomenclature variations can subtly influence the perceived completeness of collaboration networks while leaving the dominance of core institutions unaffected.

### 3.5 Keyword analysis

Leveraging the WOSCC and Scopus datasets, this study conducted a comprehensive structural and hotspot analysis of keywords using CiteSpace and R packages to explore the evolutionary trajectory and emerging trends in the field. Through co-occurrence, burst, and trend analyses, we mapped the knowledge structure and dynamic development patterns of research on *S. aureus* immune evasion.

**Figure 6:**
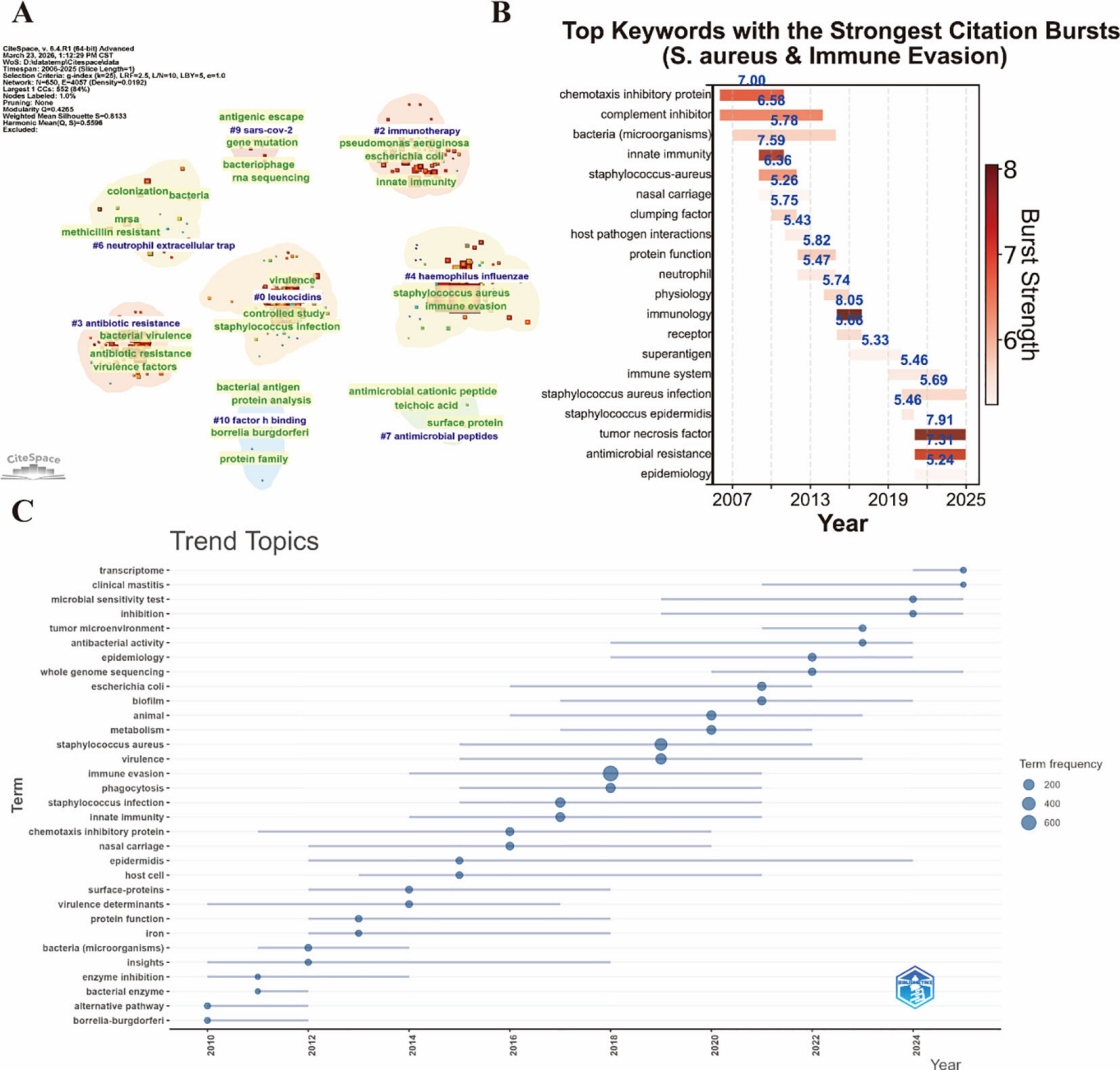
Keyword structure and identification of research hotspots. **(A)** High-frequency keyword clustering network generated by CiteSpace. **(B)** Top 20 keywords with the strongest citation bursts from 2006 to 2025, indicating shifts in research focus (e.g., “immune evasion,” “antimicrobial resistance”). **(C)** Keyword trend analysis illustrating the evolution of terms over time, transitioning from early focuses like “nasal carriage” to recent topics such as “whole-genome sequencing” and “transcriptomics.”.

High-frequency keyword clustering revealed the primary thematic modules within the field (**Figure 6A**). “*Staphylococcus aureus*” (frequency = 687) and “Immune evasion” (frequency = 645) appear to constitute the foundational pillars of this research domain (**Supplementary Table 4**). Several prominent clusters emerged around these core terms: #6 Neutrophil Extracellular Trap (NETs) included central keywords such as “Colonization,” “Bacteria,” “methicillin-resistant *Staphylococcus aureus* (MRSA)” (frequency = 91), and “Methicillin resistant,” reflecting bacterial colonization and resistance mechanisms. #3 Antibiotic Resistance encompassed “Bacterial virulence” (frequency = 114), “Antibiotic resistance” (frequency = 138), and “Virulence factors” (frequency = 19), highlighting antimicrobial resistance and virulence determinants. #0 Leukocidins featured “Virulence” (frequency = 159), “Staphylococcus infection” (frequency = 229), and “Controlled study” (frequency = 150), relating to pathogenic mechanisms and infection control. #4 *Haemophilus influenzae* reinforced the centrality of “*Staphylococcus aureus*” and “Immune evasion” through immune evasion and pathogen interaction studies. #2 Immunotherapy contained “*Escherichia coli*” (frequency = 79), “Innate immunity” (frequency = 130), and “*Pseudomonas aeruginosa*,” representing innate immunity and therapeutic intervention research. #9 SARS-CoV-2 involved terms such as “Antigenic escape,” “Gene mutation,” “Bacteriophage,” and “RNA-Sequencing,” reflecting emerging contemporary hotspots. #7 Antimicrobial Peptides included “Antimicrobial cationic peptide,” “Surface protein,” and “Teichoic acid,” highlighting novel antimicrobial strategies. #10 Factor H Binding featured “Bacterial antigen,” “Protein analysis,” “Borrelia burgdorferi,” and “Protein family,” focusing on host-pathogen interactions. The clustering network demonstrated substantial structural validity, with a modularity (Q) of 0.4265, indicating strong internal cohesion and clear separation between clusters, and an average silhouette value (S = 0.8133), supporting high intra-cluster homogeneity and overall reliability.

Temporal burst and trend analyses further illuminated the evolving research foci (**Figures 6B, C**). In the early phase (approximately 2006–2015), foundational studies appear to have concentrated on molecular mechanisms of immune evasion, with keywords such as “chemotaxis inhibitory protein” (burst strength = 7.00) and “bacteria (microorganisms)” (5.78) showing prominent bursts. The mid-phase (circa 2012–2018) shifted toward host-pathogen interactions, marked by significant bursts in “host pathogen interactions” (5.43), “protein function” (5.82), “immunology” (8.05), and “receptor” (5.66), reflecting deeper investigations into molecular interaction networks. In the later phase (2019–2024), emerging terms included “*Staphylococcus epidermidis*” (5.69) and “tumor necrosis factor” (7.91), alongside sustained attention to “antimicrobial resistance” (5.24), suggesting a broader focus encompassing commensal microbiota competition, regulation of key inflammatory mediators, and clinical resistance challenges.

Trend analysis supports this progression (**Figure 6C**): early studies (2010–2015) emphasized “nasal carriage,” “epidemiology,” “bacterial enzyme,” and “bacteria (microorganisms)”; mid-phase research (2015–2021) focused on “innate immunity,” “immune evasion,” and “phagocytosis”; and recent studies increasingly adopt omics-driven approaches, including “whole genome sequencing,” “microbial sensitivity tests,” and “transcriptome,” consistent with mechanistic investigations into antimicrobial resistance as a central frontier of current and forthcoming research.

### 3.6 Citation analysis

**Figure 7:**
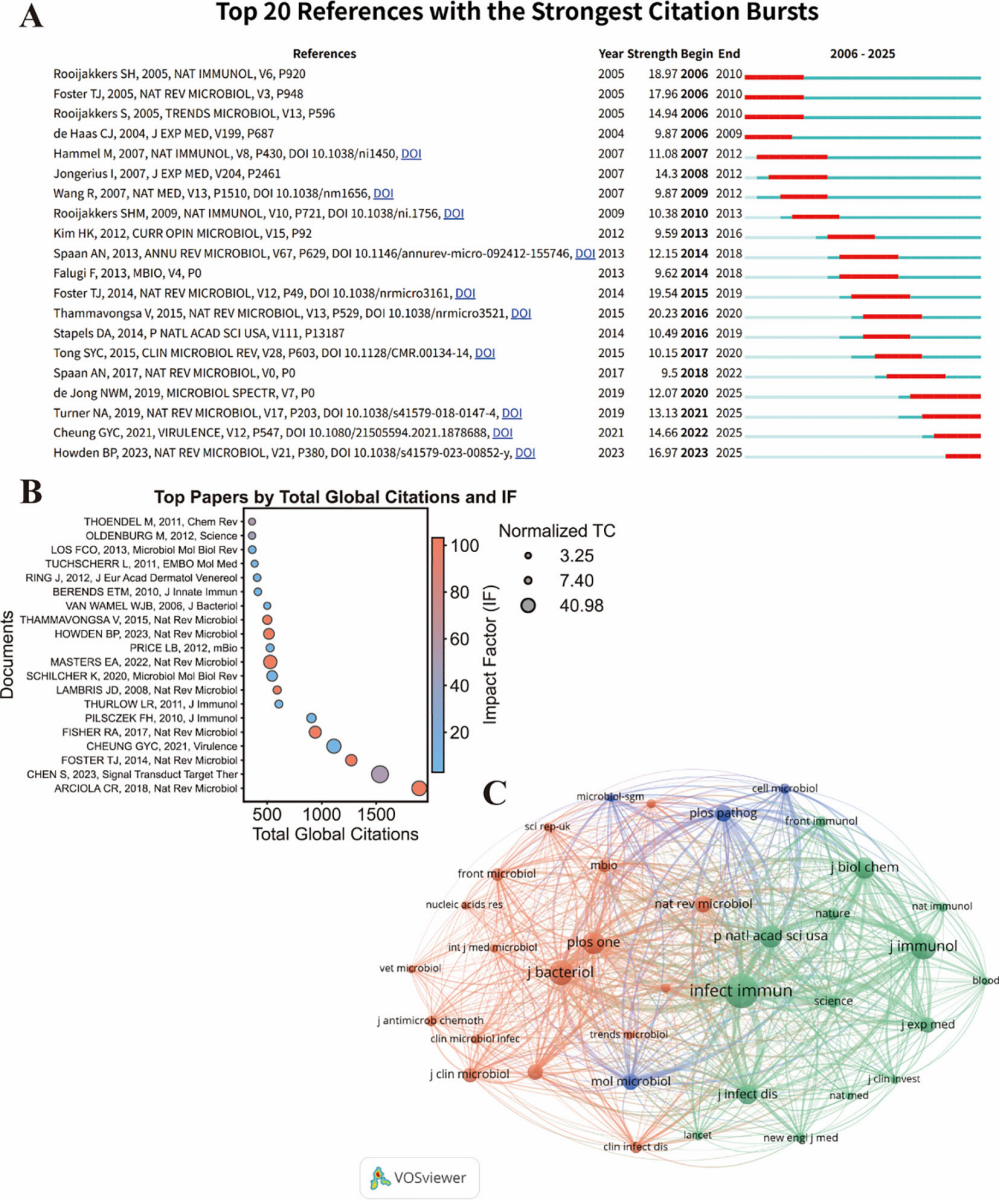
Citation analysis and knowledge structure. **(A)** Top 20 references with the strongest citation bursts. **(B)** Bubble plot of the top 20 globally cited documents, displaying citation counts and Impact Factors (IF). **(C)** Journal co-citation network visualized using VOSviewer (threshold = 400). Nodes represent journals, and links represent co-citation relationships. This network underscores the multidisciplinary nature of the field, centering on immunology and microbiology.

Citation analysis serves as a pivotal approach for elucidating the foundational knowledge and scholarly influence within a research domain. Using CiteSpace, citation burst analysis was performed to trace the dynamic evolution of research hotspots in the field of *S. aureus* immune evasion (**Figure 7A**). The top 20 references exhibiting the strongest citation bursts between 2006 and 2025 are highlighted, with the red burst segments indicating distinct temporal foci.

In the early phase (2006–2010), citation bursts appear to have primarily focused on the molecular mechanisms by which *S. aureus* interferes with the host innate immune system. Notable early burst publications include Rooijakkers SH (2005) on complement inhibitory proteins (burst strength = 18.97) and Foster TJ (2005) providing a panoramic review of *S. aureus* immune evasion (burst strength = 17.96) (20, 21).

During the mid-phase (2010–2015), research emphasis shifted toward specific virulence factors and clinical interventions. Hammel M (2007) investigated the structure of *S. aureus* Protein A (SpA) (burst strength = 11.08), elucidating how bacterial extracellular fibrinogen-binding protein (Efb-C) inhibits complement component C3 (22). Wang R (2007) explored epigenetic regulation of immune evasion (burst strength = 9.87), providing insights into why community-acquired MRSA (CA-MRSA) causes severe infections in otherwise healthy populations, contrasting with hospital-acquired MRSA that predominantly affects immunocompromised patients (23).

In the recent phase (2015–2023), citation bursts reflect a pronounced translational and clinical orientation. Thammavongsa V (2015) offered a comprehensive review of *S. aureus* as a commensal of the human nasal cavity and skin, detailing mechanisms underlying recurrent soft tissue and bloodstream infections (burst strength = 20.23, the highest in **Figure 7A**) (24). Turner NA (2019) focused on MRSA epidemiology, genetic diversity, and evolving therapeutic strategies (burst strength = 13.13), emphasizing ongoing clinical challenges and the urgent need for novel antimicrobials and adjunctive therapies (25). More recently, Howden BP (2023) represents the latest burst, reflecting a shift toward host-pathogen interaction mechanisms, with particular attention to neutrophil-mediated immune regulation, immune evasion strategies, and host-pathogen metabolic interplay (26).

Among the top 20 most-cited references, Nature Reviews Microbiology contributed seven articles, underscoring its prominence in the *S. aureus* research landscape (**Figure 7B**; **Table 5**). Arciola et al. (2018) on biofilm-associated infections leads the list with 1,895 global citations, systematically detailing biofilm formation, immune evasion, and persistence in device-related infections (27). Foster (2014) and Fisher RA (2017) provided comprehensive reviews on surface protein-mediated virulence and the role of bacterial persisters in chronic infections, respectively (28, 29) (**Table 5**). Despite differing publication years, these works exhibit high normalized total citations (e.g., Chen, 2023, achieving a normalized TC of 40.98 within a single year). High-impact references predominantly focus on “immune evasion” (e.g., Arciola CR, 2010) and “virulence factors and pathogenesis” (e.g., Foster, 2014), indicating that mechanistic exploration of pathogenesis remains the most stable knowledge core in the field.

**Table 5:** Ranking and detailed information of the top 20 globally cited documents (WOSCC+SCOPUS).

Paper	DOI	Global total citations	Normalized TC	IF
ARCIOLA CR, 2018, *Nat Rev Microbiol*	10.1038/s41579-018-0019-y	1895	30.23	103.3
CHEN S, 2023, *Signal Transduct Target Ther*	10.1038/s41392-023-01452-1	1535	40.98	52.7
FOSTER TJ, 2014, *Nat Rev Microbiol*	10.1038/nrmicro3161	1272	16.00	103.3
CHEUNG GYC, 2021, *Virulence*	10.1080/21505594.2021.1878688	1112	27.30	5.3
FISHER RA, 2017, *Nat Rev Microbiol*	10.1038/nrmicro.2017.42	941	18.35	103.3
PILSCZEK FH, 2010, *J Immunol*	10.4049/jimmunol.1000675	906	9.70	3.4
THURLOW LR, 2011, *J Immunol*	10.4049/jimmunol.1002794	607	5.47	3.4
LAMBRIS JD, 2008, *Nat Rev Microbiol*	10.1038/nrmicro1824	592	6.08	103.3
SCHILCHER K, 2020, *Microbiol Mol Biol Rev*	10.1128/MMBR.00026-19	545	13.10	7.8
MASTERS EA, 2022, *Nat Rev Microbiol*	10.1038/s41579-022-00686-0	528	23.65	103.3
PRICE LB, 2012, *mBio*	10.1128/mBio.00305-11	527	5.86	4.7
HOWDEN BP, 2023, *Nat Rev Microbiol*	10.1038/s41579-023-00852-y	516	13.77	103.3
THAMMAVONGSA V, 2015, *Nat Rev Microbiol*	10.1038/nrmicro3521	501	8.72	103.3
VAN WAMEL WJB, 2006, *J Bacteriol*	10.1128/JB.188.4.1310-1315.2006	500	3.52	2.9
BERENDS ETM, 2010, *J Innate Immun*	10.1159/000319909	415	4.44	2.9
RING J, 2012, *J Eur Acad Dermatol Venereol*	10.1111/j.1468-3083.2012.04635.x	408	4.54	8
TUCHSCHERR L, 2011, *EMBO Mol Med*	10.1002/emmm.201000115	385	3.47	8.3
LOS FCO, 2013, *Microbiol Mol Biol Rev*	10.1128/MMBR.00052-12	363	4.94	7.8
OLDENBURG M, 2012, *Science*	10.1126/science.1220363	361	4.02	45.7
THOENDEL M, 2011, *Chem Rev*	10.1021/cr100370n	361	3.25	55.7

Journal co-citation analysis further provides evidence for a multidisciplinary academic ecosystem centered on immunology and microbiology (**Figure 7C**). Infection and Immunity rank first with 3,068 citations and a total link strength of 162,928, highlighting its pivotal role in connecting diverse research communities. These are followed by The Journal of Immunology (2,132 citations) and Journal of Bacteriology (1,959 citations), which together constitute the core publication cluster in this domain. Open-access, multidisciplinary journals such as PLOS ONE and Scientific Reports also occupy key positions, reflecting both the volume and diversity of research outputs. Although Nature and Science (736 and 730 citations, respectively) do not appear in the top ten, selective publications such as Oldenburg (2012) have nonetheless catalyzed critical breakthroughs (**Table 5**; **Supplementary Table 5**). Collectively, citation analysis highlights a strong reliance on leading reviews in Nature Reviews Microbiology, while detailed mechanistic studies primarily draw from specialized immunology and microbiology journals (**Figure 7C**).

### 3.7 Thematic evolution and trend analysis

**Figure 8:**
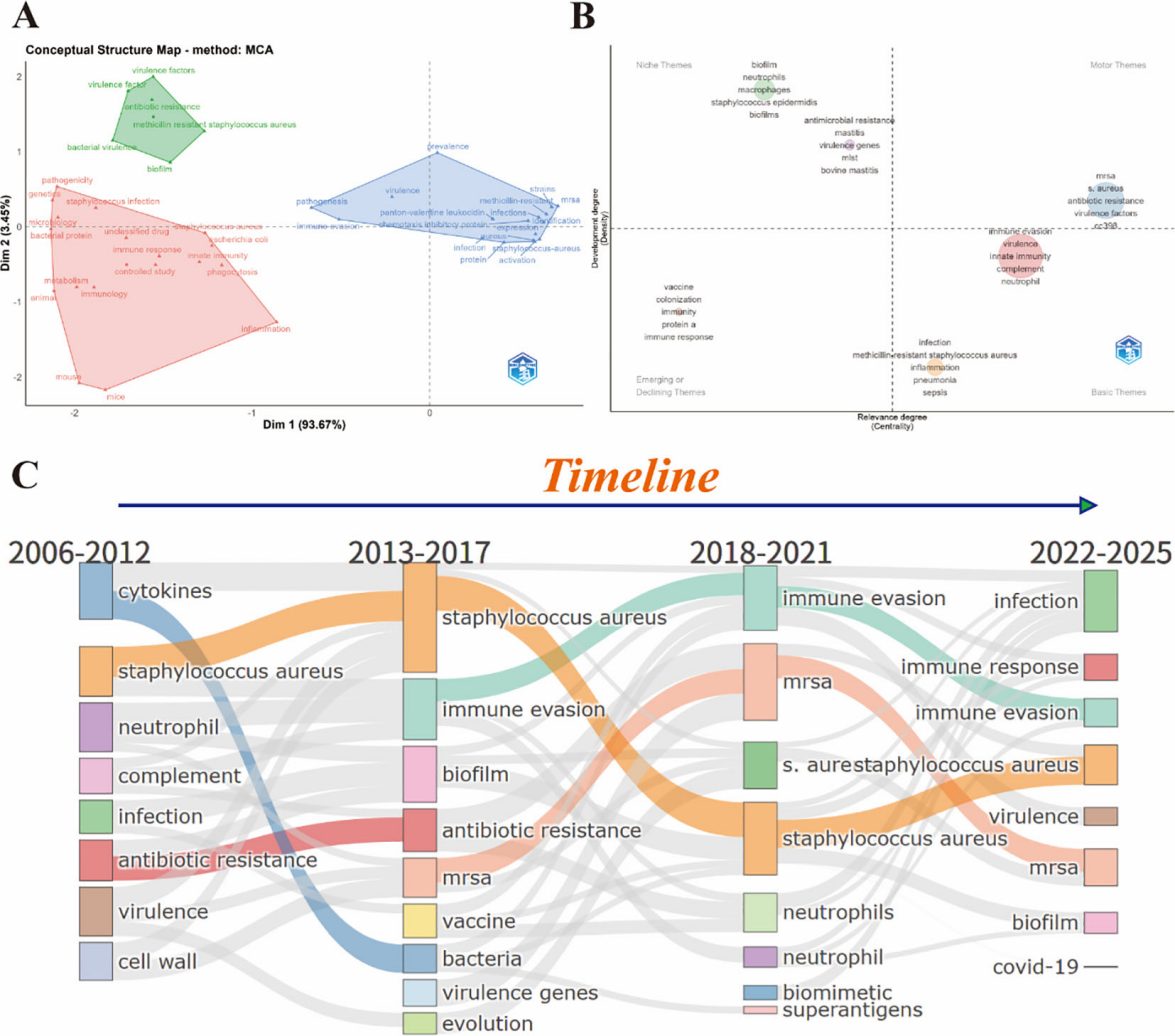
Thematic evolution and trend analysis. **(A)** Multiple Correspondence Analysis (MCA) map categorizing research themes into three clusters: green (antibiotic resistance/biofilms), blue (pathogenicity/prevalence), and red (cytokines/innate immunity). **(B)** Thematic strategic diagram (centrality vs. density), classifying themes into “Basic Hotspots,” “Driving Themes,” “Specialized Niches,” and “Emerging/Declining Themes.” **(C)** Timeline view of thematic evolution from 2006 to 2025, demonstrating a shift from basic mechanisms toward clinical translation and immunometabolism.

To explore the thematic structure and dynamic evolution of *S. aureus* research, we performed an integrated analysis combining multiple correspondence analysis (MCA), thematic centrality assessment, and timeline visualization (**Figure 8**).

The MCA revealed three distinct clusters within the two-dimensional space (**Figure 8A**). The green cluster, encompassing keywords such as antibiotic resistance, virulence factors, biofilm, and MRSA, indicates that antimicrobial resistance, biofilm formation, and virulence mechanisms constitute the current research core, forming a tightly interconnected axis central to addressing clinical infection challenges. The blue cluster, focused on pathogenicity, prevalence, and pathogenesis, reflects sustained attention to the fundamental biological characteristics of *S. aureus* and its disease progression, representing foundational research for understanding bacterial pathogenesis. The red cluster, including cytokines, innate immunity, phagocytosis, and inflammation, underscores the pivotal role of host immune responses in infection, highlighting the importance of immunological perspectives in host-pathogen interaction studies.

The thematic centrality-density map further categorized research themes into four types (**Figure 8B**). Themes in the lower-right quadrant, such as infection, inflammation, and pneumonia, represent “basic hotspot themes,” forming the foundational framework and connecting hubs of the discipline. The upper-right quadrant, comprising MRSA, *S. aureus*, and antibiotic resistance, represents “driving themes” with both high centrality and density; these constitute the most active and central research areas, highly interconnected with other themes, and serve as primary drivers of disciplinary development. The upper-left quadrant, including biofilm, neutrophils, and macrophages, represents “specialized niche themes” with lower centrality but deep, focused investigation within specific subdomains. The lower-left quadrant, comprising vaccine, colonization, and immunity, represents “emerging or declining themes,” characterized by low centrality and density, suggesting either nascent or slowly progressing research with limited network connectivity. Notably, this algorithmic marginalization of traditional vaccines in our strategic map (**Figure 8B**) is not merely a statistical artifact, but a mathematical reflection of real-world clinical setbacks, as several high-profile immunotherapeutic candidates have consistently failed to meet primary endpoints in phase II/III trials.

Timeline analysis further provided insights into the evolution of research themes from 2006 to 2025 (**Figure 8C**). Between 2006 and 2012, studies primarily addressed fundamental pathogenic mechanisms and host immune responses, with core keywords including cytokines, neutrophils, complement, and cell wall, emphasizing the biological basis of infection and virulence. From 2013 to 2017, attention shifted toward antimicrobial resistance and clinical challenges, with MRSA and antibiotic resistance emerging rapidly, accompanied by biofilm formation and immune evasion, while vaccine appeared briefly, reflecting an expansion from pathogenesis to resistance and prevention strategies. Between 2018 and 2021, thematic diversification intensified: immune evasion became a prominent focus, research on neutrophils and bacterial interactions continued, and methodological keywords such as biomimetic and superantigens emerged, signaling technological and experimental advances. From 2022 to 2025, the field exhibited both diversification and convergence: infection and immune response regained prominence, studies on MRSA and *S. aureus* remained stable, Coronavirus disease 2019 emerged as a novel keyword reflecting the influence of global public health events on secondary infection and immunity research, and biofilm studies continued to be active. Overall, *S. aureus* research has evolved sequentially from investigations of fundamental immune mechanisms and antibiotic resistance to explorations of immune evasion, targeted therapeutic strategies, and broader infection control, illustrating a progressively integrated and clinically relevant research trajectory.

## 4 Discussion

### 4.1 Quantitative findings from bibliometric analysis

Over the past two decades, research on *S. aureus* immune evasion appears to have exhibited a sustained and accelerating growth trend, with a progressive shift from foundational mechanistic studies toward clinical applications. Direct quantitative evidence from our merged WOSCC+Scopus dataset (n=1374) confirms an average annual growth rate of 15.71%, with a cubic polynomial regression model showing excellent goodness-of-fit (R^2^=0.8854). A particularly sharp inflection point occurred in 2019, after which annual publication output increased by 54.8% from 93 in 2019 to 144 in 2025. This quantitative trend may reflect the dual driving forces of escalating clinical challenges posed by MRSA and advances in experimental and analytical technologies. The advent of omics approaches, including whole-genome sequencing and transcriptomics, may have substantially fueled this expansion, driving a transition from conventional phenotypic observations to in-depth elucidation of molecular mechanisms.

The scholarly output appears to exhibit a highly polarized distribution, as evidenced by Lotka’s Law analysis showing 86.5% of authors contributed only a single paper, indicating that despite widespread participation, key contributions are primarily driven by a limited cohort of leading researchers and institutions. Geographically, North American and European countries appear to dominate both publication output and collaborative networks, whereas China, although ranking second in total publications, has more self-contained domestic research ecosystems, underscoring the need to further strengthen global research partnerships.

Analysis of the knowledge structure directly derived from keyword clustering and burst detection reveals that the current research core encompasses immune evasion mechanisms, antimicrobial resistance, and immunotherapeutic strategies. These domains are tightly interconnected through elements such as neutrophils and bacterial virulence factors, likely constituting the principal intellectual backbone of the field. The thematic structure identified in this study primarily reflects the development context of *S. aureus* immune evasion research, rather than a broader context of pathogen immune evasion research. Burst keyword analysis further highlights a temporal evolution of research focus: from early studies on immune evasion molecules, to mid-phase investigations of host-pathogen interactions, and more recently to multidimensional aspects including immune metabolism and microbial competition, reflecting a progressive orientation toward systems biology and clinical translation.

Citation and journal analyses highlight the likely complementary roles of open-access platforms (e.g., Frontiers, PLOS) in broad knowledge dissemination alongside traditional high-impact journals (e.g., Nature Reviews Microbiology), which continue to lead scholarly discourse in this domain. This multi-tiered publication landscape has facilitated both widespread knowledge propagation and rapid scholarly renewal.

Collectively, research on *S. aureus* is evolving from a primary focus on fundamental immune mechanisms toward integrative omics approaches and clinical applications. Future investigations are expected to prioritize elucidation of resistance mechanisms, modulation of immune metabolism and inflammatory responses, and the development of immunotherapeutic and precision anti-infective strategies, thereby fostering interdisciplinary integration and enhancing translational impact.

### 4.2 Independent clinical trial analysis to validate bibliometrically identified translational gaps

We performed an integrative analysis of clinical trial data on *S. aureus* immunotherapy independently retrieved from PubMed, which was not included in the primary WOSCC and Scopus bibliometric datasets. Applying rigorous inclusion and exclusion criteria (**Figure 1**; **Supplementary Table 2**), a total of 10 Phase II/III clinical trials evaluating monoclonal antibodies and vaccines against *S. aureus* were included in the final analysis (**Table 6**). Overall, although immunotherapeutic strategies against *S. aureus* have shown theoretical promise in early-stage investigations, all 10 included clinical trials of most candidate interventions failed to achieve prespecified clinical endpoints, suggesting a profound translational gap between preclinical success and clinical efficacy.

**Table 6:** Integrated analysis of S. aureus immunotherapy clinical trials from PubMed.

PMCID	Investigational product	Protein (antigen)	Study population	Study design	Key findings
16870768	Tefibazumab	Humanized monoclonal antibody (ClfA)	Adults with *S. aureus* bacteremia	Single intravenous dose of Tefibazumab 20 mg/kg vs placebo, both with standard of care (SoC)	No difference in composite clinical outcome: 6.7% (2/30) vs 13.3% (4/30) (P=0.455)
17893153	AltaStaph	Polyclonal human IgG (CP5 and CP8)	Patients ≥7 years with *S. aureus* bacteremia	AltaStaph 200 mg/kg IV twice vs placebo, both with SoC	No difference in time to bacteremia clearance: median 1 day vs 2 days (P=0.58); fever resolution: 2 vs 3 days (P=0.3)
16598296	AltaStaph	Polyclonal human IgG (CP5 and CP8)	Very low birth weight infants	AltaStaph 1,000 mg/kg IV twice vs placebo	Identical incidence of *S. aureus* bacteremia: 18.8% vs 18.8%
17719934	Veronate	Donor-derived human antibodies (ClfA)	Very low birth weight infants	Veronate 750 mg/kg IV daily (up to 4 doses) vs placebo	No difference in late-onset sepsis: 5% (50/989) vs 6% (60/994) (P=0.34)
21788224	Pagibaximab	Chimeric mouse–human monoclonal antibody (lipoteichoic acid)	Very low birth weight infants	Pagibaximab 90 mg/kg or 60 mg/kg IV weekly (3 doses) vs placebo	Confirmed *S. aureus* sepsis: 0% (high dose) vs 20% (low dose) vs 13% (placebo) (P<0.11)
33894131	Suvratoxumab	Monoclonal antibody (Hla)	Mechanically ventilated ICU patients	Single IV dose of 2,000 mg or 5,000 mg vs placebo	No difference in *S. aureus* pneumonia: 18% vs 26% (RR reduction 31.9%; 90% CI, −7.5 to 56.8)
11844850	StaphVAX	Bivalent conjugate vaccine (CP5 and CP8)	Adults undergoing hemodialysis	StaphVAX vs placebo	No difference in bacteremia incidence: efficacy 26% (P=0.23); *post hoc* at 40 weeks: 57% (P=0.02)
23549582	V710	Monovalent vaccine (IsdB)	Adults undergoing median sternotomy cardiac surgery	V710 vs placebo	No difference in bacteremia or deep infection: 2.6 vs 3.2 per 100 person-years; RR 0.81 (95% CI, 0.44–1.48)
33962841	NDV-3A	Monovalent vaccine (N-terminal fragment of Candida albicans Als3)	Military recruits aged 17–42	NDV-3A vs placebo	No difference in colonization: 25.6% vs 29.1%; efficacy 12.1% (P=0.31)
37125490	SA4Ag	Quadrivalent conjugate vaccine (CP5, CP8, ClfA, rP305A)	Adults undergoing elective spinal instrumentation surgery	SA4Ag vs placebo	No difference in bacteremia or deep infection: 0.9% vs 0.9%; efficacy 0% (95% CI, −126.3 to 55.8)

Data were independently retrieved and screened from PubMed. Only 10 studies met the strict inclusion criteria. This table summarizes antigen targets (e.g., ClfA, α-hemolysin) and clinical outcomes. The consistent failure of monoclonal antibodies and vaccines in Phase II/III trials highlights the translational gap between mechanistic research and clinical application.

A detailed examination of clinical trial outcomes reveals that interventions targeting distinct antigenic epitopes of *S. aureus* virulence factors have encountered substantial obstacles. Tefibazumab, a monoclonal antibody against ClfA, did not improve composite clinical outcomes in bacteremia trials, demonstrating no statistically significant difference compared with placebo (*P* = 0.455) (30). Similarly, the polyclonal antibody AltaStaph, directed against CP5 and CP8 antigens, failed to accelerate bacteremia clearance or reduce fever duration relative to placebo (31, 32). Suvratoxumab, targeting α-hemolysin, also did not significantly reduce the incidence of pneumonia among mechanically ventilated Intensive Care Unit (ICU) patients (33).

In the vaccine domain, the IsdB-targeted V710 vaccine did not lower infection risk in patients undergoing cardiac surgery (RR 0.81) (34). The CP5/CP8-conjugated StaphVAX showed a modest long-term protective trend in *post hoc* analyses among dialysis patients, yet primary endpoints were not statistically significant (35). The quadrivalent SA4Ag vaccine, which included CP5, CP8, ClfA, and rP305A, exhibited no preventive efficacy in patients undergoing spinal surgery (0%; 95% CI, −126.3 to 55.8) (36). Additionally, the NDV-3A vaccine, designed to prevent nasal colonization, did not significantly reduce colonization rates in young military recruits (*P* = 0.31) (37).

These findings emphasize that the translational pathway for *S. aureus* immunotherapy from bench to bedside remains highly challenging. Current strategies, which often rely on single antigens or limited antigenic combinations, are insufficient to address the bacterium’s complex pathogenic mechanisms and the heterogeneous human immune environment.

Our bibliometric analysis outlines three distinct stages of research on *S. aureus* immune evasion over the past two decades. The first stage, spanning from 2006 to 2015, concentrated on the molecular mechanisms of individual virulence factors. This was followed by a shift in focus to host-pathogen interaction networks from 2012 to 2018. Most recently, from 2019 to the present, there have been rapid advancements in understanding antibiotic resistance, biofilm biology, and immunometabolic regulation. Importantly, nearly all completed immunotherapy trials have utilized the single-antigen strategy from the first stage, which may contribute to a clear temporal and conceptual gap that helps explain their widespread failure to achieve primary clinical endpoints.

Keyword analyses demonstrate that *S. aureus* possesses a highly redundant and synergistic immune evasion system featuring mechanisms such as SpA-mediated antibody neutralization, leukocidin-induced immune cell lysis, biofilm protective barriers, and lactate-driven epigenetic regulation. These complexities may undermine the effectiveness of single-target strategies (e.g., tefibazumab, suvratoxumab, and V710). Furthermore, thematic evolution analysis reveals that research on biofilms began to gain prominence only after 2013, yet all completed trials have primarily concentrated on acute planktonic infections, neglecting the clinically significant chronic biofilm-associated diseases.

Thematic mapping further classifies vaccine and immunotherapy themes within the “emerging/declining” quadrant, indicating a lack of sufficient foundational depth to facilitate successful translation into clinical practice. This translational gap is further compounded by biological and methodological challenges, including *S. aureus*-induced immune tolerance, heterogeneous immune responses among high-risk populations, and inadequate representation of human infection scenarios in conventional animal models.

Future research may prioritize the development of multivalent combinatorial therapeutics, such as antibody-drug conjugates and anti-biofilm agents, to address intracellular bacteria and chronic infections. Additionally, efforts will be directed toward optimizing vaccine adjuvants and delivery systems to balance humoral and cellular immunity while overcoming immune imprinting. Other key priorities may include refining animal models, adopting stratified clinical trial designs, investigating combination regimens of vaccines, monoclonal antibodies, and antibiotics, as well as identifying novel targets through reverse vaccinology, multispecific antibodies, and other innovative technologies.

### 4.3 Targeted mechanistic interpretation of bibliometric hotspots

#### 4.3.1 Disruption of immune surveillance and cytotoxicity

Our bibliometric analysis identified “disruption of immune surveillance” as the dominant research theme during the 2006-2015 period, with “Protein A” (frequency=87), “TLR-2” (frequency=62), and “leukocidins” (frequency=59) ranking among the top 20 most frequent keywords. The keyword “chemotaxis inhibitory protein” exhibited the strongest citation burst in this period (burst strength=7.00).

The molecular basis for this theme lies in *S. aureus*’s ability to actively interfere with multiple steps of the host immune response. *S. aureus* evades host immune defenses by producing a wide array of virulence factors that interfere with immune recognition and compromise the function of critical defense cells (38, 39). Surface-expressed Spa binds specifically to the Fcγ region of antibodies, thereby preventing effective antigen engagement and evading opsonophagocytosis (40, 41). In addition to this, SpA disrupts B cell receptor signaling, leading to immunosuppressive effects that hinder the activation and proliferation of adaptive immune cells (42).

**Figure 9:**
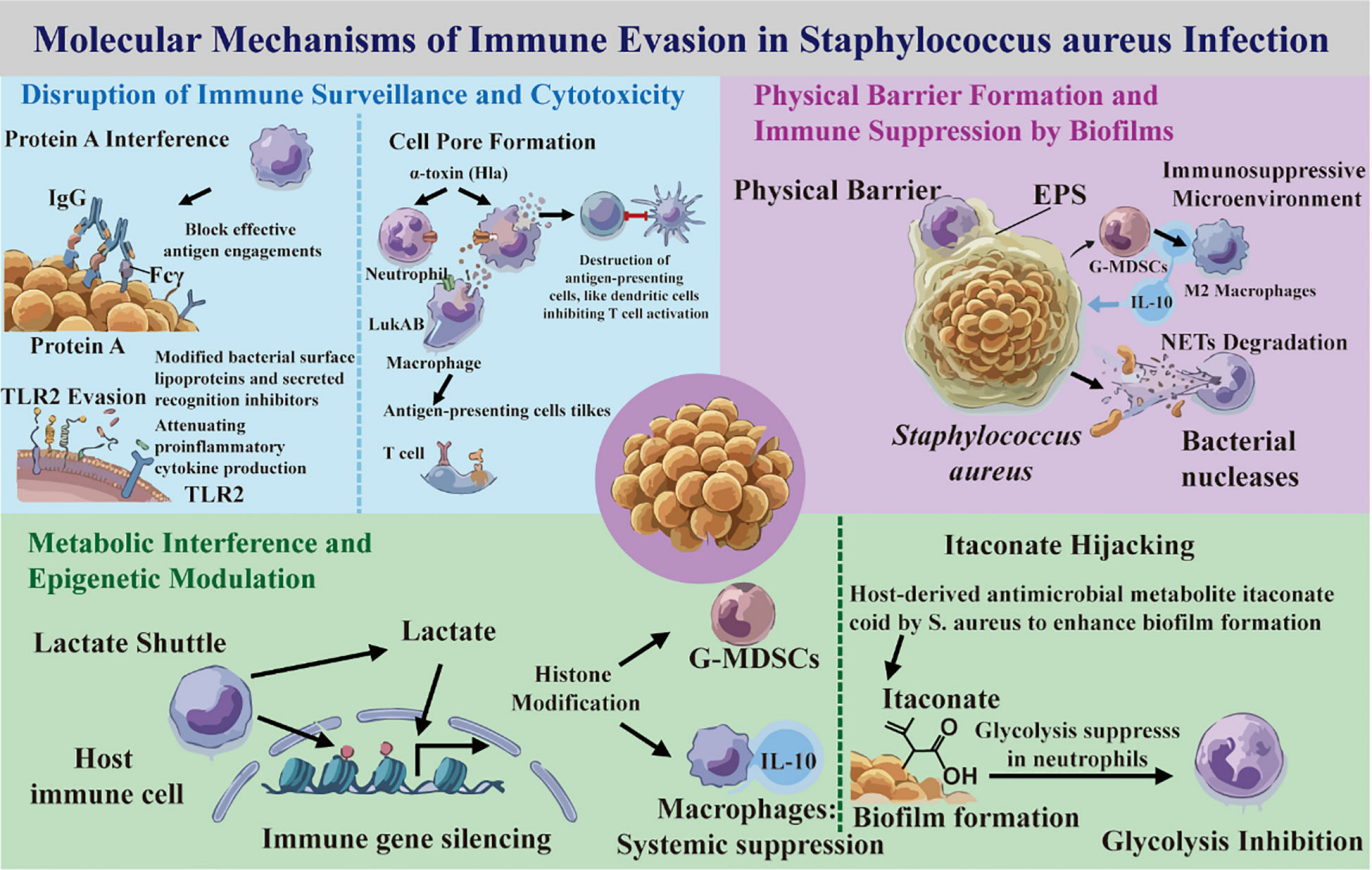
Schematic of molecular mechanisms of immune evasion in *S. aureus* infection. This diagram summarizes three core immune evasion strategies employed by *S. aureus*: (1) Disruption of immune surveillance, where the bacterium utilizes Protein A to bind Fcγ receptors and block antibody function, interferes with TLR-2 signaling, and secretes cytotoxins (e.g., α-toxin, leukocidins) to induce immune cell lysis; (2) Construction of an immunosuppressive microenvironment via biofilm formation (physical barrier) and the induction of myeloid-derived suppressor cell (MDSC) expansion and IL-10 secretion; and (3) Metabolic and epigenetic regulation, utilizing metabolites such as lactate and itaconate to alter host immunometabolism and induce immune tolerance, thereby facilitating persistent infection.

Moreover, *S. aureus* modifies its surface lipoproteins or secretes recognition inhibitors to escape detection by TLR-2, attenuating proinflammatory cytokine production and rendering the early stages of infection less detectable to the host immune system (43). Secreted cytotoxins, including α-toxin (Hla) and leukocidins such as LukAB, create pores in the membranes of neutrophils, macrophages, and other immune cells, causing cell lysis and functional impairment (44, 45). In addition, the destruction of antigen-presenting cells such as dendritic cells compromises antigen presentation, inhibits T cell activation, and undermines adaptive immunity (46, 47). Collectively, these strategies enable *S. aureus* to subvert both innate and adaptive immune defenses, potentially establishing conditions favorable for persistent infection and systemic dissemination (**Figure 9**).

#### 4.3.2 Biofilm-mediated immune evasion

Biofilm research appears to have emerged as a distinct core cluster in our keyword analysis (Cluster #6, modularity Q=0.4265) and has demonstrated significant growth since 2013. The term “biofilm” has accumulated a frequency of 114, while “NETs” has shown a notable citation burst beginning in 2017 (burst strength=5.33).

In the context of chronic or device-associated infections, *S. aureus* establishes a robust dual-layered defense through biofilm formation (48). Biofilms, which consist of dense extracellular polymeric substances (EPS), physically impede immune cell infiltration and phagocytosis, thereby providing a protective niche for bacterial survival (49).

Beyond this physical barrier, the biofilm microenvironment actively modulates host immunity by promoting the accumulation of granulocytic myeloid-derived suppressor cells (G-MDSCs) and anti-inflammatory macrophages. These cells not only exhibit reduced bactericidal function but also secrete immunosuppressive factors that inhibit T cell activation, further dampening host immune responses (50, 51). In parallel, bacterial nucleases degrade NETs, disrupting host mechanisms for capturing and killing bacteria, which facilitates long-term colonization and persistence (52, 53). Thus, biofilms provide both a physical shield and an immunosuppressive niche, enabling *S. aureus* to evade immune clearance effectively (**Figure 9**).

Importantly, this microenvironmental suppression may explain why advanced monoclonal antibodies (such as Suvratoxumab) show limited efficacy in biofilm-rich clinical settings (33). Within the biofilm matrix, *S. aureus* actively reprograms the host immune landscape, driving glycolysis and the hypoxia response through a hypoxia-inducible factor-1α axis in infiltrating G-MDSCs (51). This metabolic hijacking forces host myeloid cells into an anti-inflammatory, IL-10-producing phenotype, completely paralyzing the cellular phagocytosis required for antibody-mediated clearance. Thus, even when therapeutic antibodies successfully penetrate the physical barrier, their efficacy may be influenced by the absence of active, healthy effector cells in the metabolic microenvironment (50, 51).

#### 4.3.3 Metabolic interference and epigenetic modulation

Immunometabolism appears to be the fastest-growing research area in our dataset, with publication output increasing by 320% between 2019 and 2025. The keyword “tumor necrosis factor” exhibited the strongest citation burst in the entire study period (burst strength=7.91), and “IL-10” (frequency=52) showed a 4.1-fold increase in occurrence rate after 2020.

*S. aureus* appears to have evolved sophisticated mechanisms to manipulate host immune metabolism to induce immune tolerance and facilitate persistence. Lactate produced within the biofilm penetrates host immune cells and acts as a signaling molecule that triggers epigenetic modifications, including histone modifications. These changes stimulate G-MDSCs and macrophages to secrete the anti-inflammatory cytokine IL-10, resulting in systemic suppression of bactericidal immunity (11, 54).

In certain infection contexts, such as pneumonia, host-derived antimicrobial metabolites like itaconate, which normally inhibit bacterial growth, are co-opted by *S. aureus* to enhance biofilm formation. Concurrently, itaconate suppresses glycolysis in neutrophils, reducing their energy supply and diminishing their bactericidal capacity (55, 56). Through these metabolic and epigenetic strategies, *S. aureus* effectively modulates host immune responses to create a permissive environment for chronic infection, immune evasion, and prolonged colonization (**Figure 9**).

### 4.4 Integrated perspectives and future directions

Despite ongoing advances in antimicrobial development, *S. aureus* continues to rank among the leading causes of infection-related mortality, with persistently high case-fatality rates observed in bacteremia and infective endocarditis (57). The field faces two interconnected core challenges, namely the relentless spread of antimicrobial resistance and the profound translational gap between preclinical discoveries and clinical interventions, confirmed by our summary of 10 Phase II/III immunotherapy trials. We synthesize current clinical progress and propose three priority research directions to address these unmet needs.

#### 4.4.1 Current management and treatment of *S. aureus* infections

A critical clinical challenge remains the insufficient capacity for early and accurate diagnosis. Future improvements may be achieved through the integration of advanced imaging techniques with next-generation sequencing of microbial cell-free DNA (cfDNA), which could enable rapid, precise localization of infection foci, facilitate timely therapeutic interventions, and enhance patient outcomes (58, 59).

From a therapeutic perspective, traditional fixed-duration antibiotic regimens fail to account for the heterogeneity of individual patient responses. Emerging strategies are increasingly focused on personalized therapy guided by dynamic biomarkers and artificial intelligence-driven algorithms, which allow real-time monitoring of pathogen clearance. This approach can optimize treatment duration on a patient-specific basis, which may reduce the risks associated with both under-treatment and overtreatment (60–62).

At the pharmacologic level, fifth-generation cephalosporins (i.e., ceftobiprole and ceftaroline) and long-acting lipoglycopeptides (dalbavancin) provide promising options for the management of MRSA infections. However, evidence from clinical trials including CAMERA-2 and ARREST suggests that conventional combination antibiotic therapy has not achieved a significant reduction in mortality (63, 64). In contrast, the POET and SABATO studies demonstrate that, in rigorously selected low-risk patients, sequential oral therapy is non-inferior to continuous intravenous therapy. These findings highlight a potential future trend toward treatment de-escalation and oral transition strategies, which balance therapeutic efficacy with patient convenience and quality of life (65, 66).

In the domain of biologics, bacteriophage therapy and related phage-derived approaches are gaining recognition as important adjunctive options. Early clinical studies indicate that phage cocktail therapy is safe and potentially effective in complex bacteremia cases (67, 68). Furthermore, the phage-derived lysin exebacase demonstrates potent bactericidal activity and favorable pharmacokinetic profiles, supporting its development for the treatment of biofilm-associated infections (69–71).

Immunotherapeutic interventions targeting key virulence mechanisms are also rapidly evolving. Multivalent monoclonal antibodies, such as mAbtyrin, are capable of neutralizing multiple virulence pathways simultaneously. Additionally, Fc-engineered antibodies designed to resist SpA-mediated immune interference show promise for prophylactic application in high-risk populations, including mechanically ventilated patients, potentially mitigating severe outcomes in critical care settings (72–74).

#### 4.4.2 Priority future research directions informed by our study

Based on an integrated analysis of bibliometric trends and clinical trial outcomes, we propose three priority research directions to address the challenges posed by *S. aureus* infection. The organism’s redundant and highly adaptive immune evasion mechanisms may render single-antigen vaccines and monoclonal antibodies largely ineffective. Therefore, it may be crucial to move away from single-target strategies and adopt combination therapies that concurrently disrupt multiple virulence pathways, prevent biofilm formation, and modulate host immune responses. Among emerging fields, immunometabolism shows great promise, evidenced by a remarkable increase in related publications since 2019. Targeting the bidirectional metabolic crosstalk between bacteria and the host may present a novel approach to managing antibiotic-refractory chronic infections and biofilm-associated diseases. Furthermore, we should expedite the translation of multi-omics findings to develop tailored anti-infective regimens. This entails integrating pathogen genomic data, host immunometabolic profiles, and clinical biomarkers to create precision therapies that enhance treatment outcomes against *S. aureus* infections.

Taken together, the future management of *S. aureus* infections is likely to integrate precision diagnostics, personalized pharmacologic regimens, innovative biologic therapies, and advanced immunotherapeutic strategies. Such a multi-faceted approach has the potential to address the complexity of bacterial pathogenesis, improve clinical outcomes, and guide the rational deployment of next-generation anti-staphylococcal interventions.

### 4.5 Limitations

Several methodological limitations should be considered when interpreting our findings. First, to enhance consistency and comparability of citation data across studies, only English-language publications were included. While this ensures standardized data and broad international readability, it may exclude research published in regional languages such as Chinese, Japanese, or Korean. Although these studies carry local academic value, their global citation impact is relatively limited, likely exerting minimal influence on overall trends.

Second, the WOSCC and Scopus differ in coverage, update mechanisms, and document-type composition. WOSCC offers a longer temporal span and a relatively stable citation framework, whereas Scopus provides broader coverage and higher update frequency. To integrate the strengths of both databases, cross-deduplication and field standardization were employed to mitigate systematic biases. Nevertheless, discrepancies in indexing rules and data structures may still introduce minor variability in institutional attribution and collaboration network construction.

Third, standardization of author and institution names is constrained by the precision of bibliometric tools, and occasional mismatches may occur. Given the large sample size and extended time span, these technical deviations are unlikely to materially alter overall trends or core conclusions. It is important to emphasize that bibliometric analysis is inherently a macro-level, retrospective approach aimed at mapping field evolution and knowledge structures rather than substituting for specific clinical or mechanistic studies. Within this framework, the present study provides a quantitative reference for understanding the landscape and developmental trajectory of *S. aureus* immune evasion research.

## 5 Conclusion

Through a comprehensive bibliometric mapping of two decades of research on *S. aureus* immune evasion, we have observed that the field is poised at a pivotal transition, moving from foundational mechanistic studies toward clinical translation. Our analysis reveals a marked polarization in scientific productivity, with leading institutions in the United States and Europe exerting dominant influence over knowledge generation and collaborative networks. Although China ranks prominently in overall publication output, we note that its contributions predominantly center on biofilm formation and antibiotic resistance surveillance rather than on pioneering theoretical frameworks.

Tracing the evolution of the knowledge structure, we find a clear trajectory: early investigations largely addressed individual virulence factors, whereas more recent studies increasingly focus on integrative analyses of host–pathogen interactions, immune-metabolic regulation, and multi-omics mechanisms. By incorporating clinical trial data from PubMed, we further underscore the persistent translational gap, as conventional vaccines and monoclonal antibodies targeting single antigens frequently fail to yield meaningful clinical benefit in the context of the bacterium’s sophisticated and redundant immune evasion strategies.

Looking forward, we advocate for a shift from broad descriptive approaches toward precise, mechanistically informed interventions. We envision that multi-targeted strategies informed by systems-level understanding will be essential to bridge the divide between experimental discovery and clinical implementation, ultimately enabling more effective therapeutic solutions against *S. aureus* infections.

## Data Availability

The raw data supporting the conclusions of this article will be made available by the authors, without undue reservation.
