# Supplementary material for: The evolving landscape of Staphylococcus aureus immune evasion: a data-driven atlas over two decades
Source: Front Immunol. 2026 Jul 16;17:1862459. doi: 10.3389/fimmu.2026.1862459 (PMC13422175; doi:10.3389/fimmu.2026.1862459)
Supplement: Supplementary file 1 [file DataSheet1.docx]

**Supplementary Materials:**

**Supplementary Table 1. Detailed literature search strategies for each database.**

| **Database** | **Search Strategy** | **Search Date** | **Filtering Conditions** |
| --- | --- | --- | --- |
| WOSCC | TS1=("Staphylococcus aureus" OR "S. aureus" OR "Staph aureus");  TS2=("immune evasion" OR "immune escape" OR "immune suppress*" OR "immune avoid*" OR "host immune response evasion");  TS=TS1 AND TS2 | The literature search was conducted on March 10, 2026. | English-language publications from January 1, 2006, to December 31, 2025, including Articles and Review Articles. |
| Scopus | ( TITLE-ABS-KEY ( ( "Staphylococcus aureus" OR "S. aureus" OR "Staph aureus" ) ) AND TITLE-ABS-KEY ( ( "immune evasion" OR "immune escape" OR "immune suppress*" OR "immune avoid*" OR "host immune response evasion" ) ) ) | The literature search was conducted on March 10, 2026. | English-language publications from January 1, 2006, to December 31, 2025, including Articles and Review Articles. |
| PubMed | ("Staphylococcus aureus"[Mesh] OR "Staphylococcus aureus"[tiab] OR staphylococcal sepsis[tiab] OR staphylococcal bloodstream infection[tiab]) AND ( "Immunotherapy"[Mesh] OR "Vaccines"[Mesh] OR "Bacterial Vaccines"[Mesh] OR "Antibodies"[Mesh] OR "Antibodies, Monoclonal"[Mesh] OR "Immunoglobulins"[Mesh] OR "monoclonal antibody"[tiab] OR "immune globulin"[tiab] OR "vaccine"[tiab] ) AND ( "Clinical Trial"[Publication Type] OR "Randomized Controlled Trial"[Publication Type] ) | The literature search was conducted on March 10, 2026. | Clinical trials and RCTs meeting eligibility criteria and included for discussion |

**Supplementary Table 2. Comparison of annual citation changes across databases (WOSCC, SCOPUS, and Merged Dataset).**

| **Year** | **WOSCC** | **Scopus** | **WOSCC+Scopus** |
| --- | --- | --- | --- |
| 2006 | 150.50 | 174.80 | 142.22 |
| 2007 | 70.44 | 98.71 | 79.08 |
| 2008 | 100.57 | 78.40 | 97.33 |
| 2009 | 83.59 | 98.57 | 87.54 |
| 2010 | 86.97 | 84.42 | 93.43 |
| 2011 | 85.87 | 112.70 | 110.97 |
| 2012 | 86.31 | 84.98 | 89.86 |
| 2013 | 77.48 | 72.85 | 73.46 |
| 2014 | 95.39 | 81.28 | 79.48 |
| 2015 | 41.94 | 61.50 | 57.49 |
| 2016 | 38.61 | 40.15 | 41.09 |
| 2017 | 50.03 | 43.23 | 51.29 |
| 2018 | 61.75 | 74.44 | 62.68 |
| 2019 | 35.12 | 40.47 | 41.56 |
| 2020 | 33.92 | 37.44 | 41.61 |
| 2021 | 48.27 | 41.96 | 40.73 |
| 2022 | 23.33 | 22.98 | 22.32 |
| 2023 | 22.43 | 41.43 | 37.46 |
| 2024 | 9.49 | 10.10 | 10.96 |
| 2025 | 3.30 | 2.65 | 3.00 |

**Supplementary Table 3. Frequency statistics of the top 30 keywords.**

| Keywords | Count |
| --- | --- |
| staphylococcus aureus | 687 |
| immune evasion | 645 |
| staphylococcus infection | 229 |
| infection | 194 |
| phagocytosis | 193 |
| virulence | 159 |
| controlled study | 150 |
| antibiotic resistance | 138 |
| innate immunity | 130 |
| microbiology | 114 |
| bacterial virulence | 114 |
| animal | 112 |
| immunology | 111 |
| immune response | 102 |
| metabolism | 98 |
| methicillin resistant staphylococcus aureus | 91 |
| genetics | 89 |
| panton valentine leukocidin | 86 |
| prevalence | 80 |
| escherichia coli | 79 |
| activation | 79 |
| identification | 78 |
| antimicrobial resistance | 70 |
| bacterial protein | 68 |
| biofilm | 64 |
| pathogenesis | 64 |
| mouse | 63 |
| mice | 62 |
| pathogenicity | 61 |
| virulence factors | 19 |

**Supplementary Table 4. Top 20 co-cited journals (WOSCC+SCOPUS).**

| source | citations | total link strength |
| --- | --- | --- |
| infect immun | 3068 | 162928 |
| j immunol | 2132 | 100443 |
| j bacteriol | 1959 | 99158 |
| p natl acad sci usa | 1717 | 91199 |
| plos one | 1713 | 83878 |
| j biol chem | 1589 | 81624 |
| j infect dis | 1418 | 80480 |
| nat rev microbiol | 1103 | 53833 |
| plos pathog | 1092 | 67254 |
| mol microbiol | 1074 | 59544 |
| antimicrob agents ch | 1013 | 50035 |
| j exp med | 1001 | 53073 |
| j clin microbiol | 979 | 36384 |
| mbio | 795 | 44803 |
| front microbiol | 791 | 36749 |
| nature | 736 | 38130 |
| science | 730 | 40440 |
| clin infect dis | 671 | 34396 |
| front immunol | 606 | 31481 |
| j antimicrob chemoth | 590 | 22670 |

**Supplementary Table 5. Inclusion and exclusion criteria for PubMed S. aureus immunotherapy clinical trials.**

| **Criteria Type** | **Details** |
| --- | --- |
| Inclusion Criteria | Clinical trials or randomized controlled trials (RCTs); participants with Staphylococcus aureus infection or at high risk of infection, including adults, children, and very low birth weight infants; interventions consisting of investigational products targeting specific S. aureus antigens (e.g., monoclonal antibodies, polyclonal immunoglobulins, or vaccines), compared with placebo or standard of care (SoC); reporting at least one clinically relevant outcome, such as composite endpoints, time to bacteremia clearance, time to fever resolution, incidence of infection, pneumonia, or colonization. |
| Exclusion Criteria | Non-randomized studies (e.g., reviews, case reports, or observational studies); populations not clearly defined as having S. aureus infection or being at increased risk; interventions not targeting specific S. aureus antigens or lacking a placebo or SoC comparator; failure to report any clinically relevant outcomes. |

**
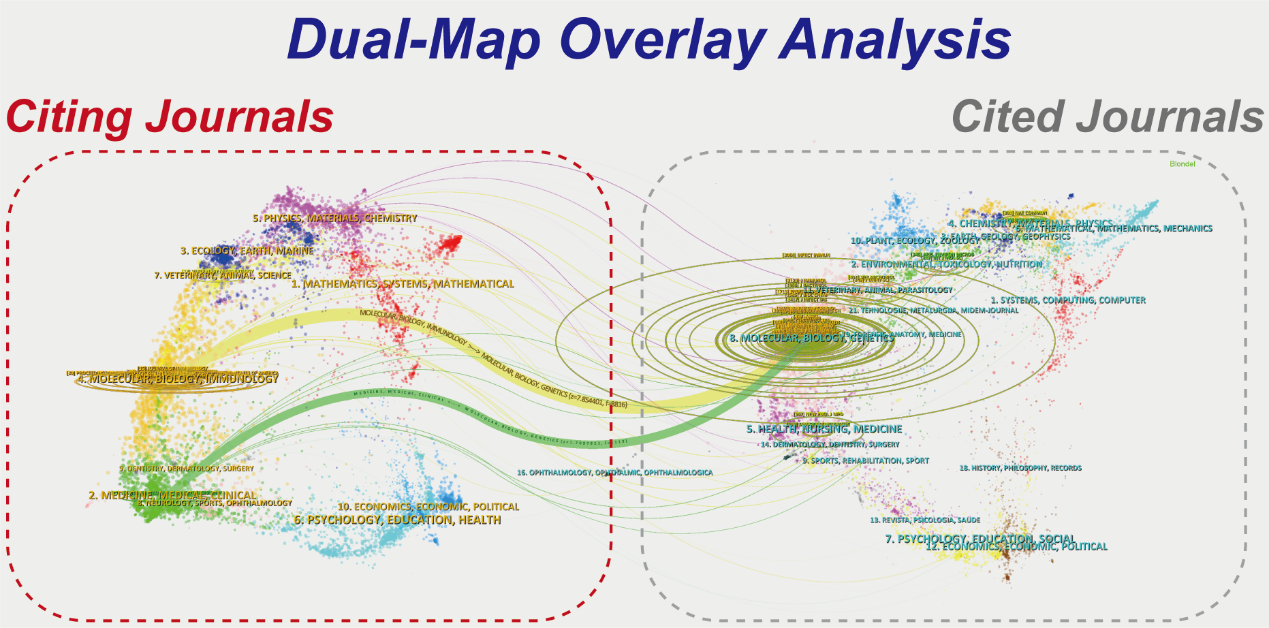
**

**Supplementary Figure 1. Dual-map overlay analysis (WOSCC+SCOPUS).**

*Note:* Visualized using CiteSpace. This figure maps the knowledge structure by displaying the overlap between citing journals (left side) and cited journals (right side). It reveals the interdisciplinary convergence between clinical medicine (e.g., Infectious Diseases) and basic biological sciences (e.g., Molecular Biology), indicating that translational research serves as a bridge connecting these fields.

| *S. aureus* | *Staphylococcus aureus* |
| --- | --- |
| TLR-2 | Toll-like receptor 2 |
| MDSCs | myeloid-derived suppressor cells |
| IL-10 | interleukin-10 |
| WoSCC | Web of Science Core Collection |
| RCTs | **randomized controlled trials** |
| MCP | **multiple country publication** |
| SCP | **single country publication** |
| TC | **Total citation** |
| NIH | **National Institutes of Health** |
| NETs | **Neutrophil Extracellular Trap** |
| MRSA | **methicillin-resistant Staphylococcus aureus** |
| SARS-CoV-2 | **Severe acute respiratory syndrome coronavirus 2** |
| Efb-C | **extracellular fibrinogen-binding protein** |
| CA-MRSA | **community-acquired MRSA** |
| IF | Impact Factors |
| MCA | multiple correspondence analysis |
| ClfA | clumping factor A |
| CP | capsular polysaccharide |
| IsdB | **iron-regulated surface determinant protein B** |
| ICU | **intensive care unit** |
| EPS | extracellular polymeric substances |
| G-MDSCs | granulocytic myeloid-derived suppressor cells |
| cfDNA | cell-free DNA |
